# CNVs in Epilepsy

**DOI:** 10.1007/s40142-014-0046-6

**Published:** 2014-06-28

**Authors:** Heather C. Mefford

**Affiliations:** Division of Genetic Medicine, Department of Pediatrics, University of Washington, RR349A, Box 356320, Seattle, WA USA

**Keywords:** Copy number variants, Epilepsy, Microdeletions, Neurodevelopmental disorders

## Abstract

Copy number variants (CNVs) are deletions or duplications of DNA. CNVs have been increasingly recognized as an important source of both normal genetic variation and pathogenic mutation. Technologies for genome-wide discovery of CNVs facilitate studies of large cohorts of patients and controls to identify CNVs that cause increased risk for disease. Over the past 5 years, studies of patients with epilepsy confirm that both recurrent and non-recurrent CNVs are an important source of mutation for patients with various forms of epilepsy. Here, we will review the latest findings and explore the clinical implications.

## Introduction

Epilepsy is a group of conditions characterized by recurrent, unprovoked seizures that result from abnormal synchronized neuronal firing in the brain. Epilepsy will affect up to 1 in 26 individuals and confers a significant health and economic burden [1]. There are many forms of epilepsy that can be distinguished by various characteristics including age of onset, predominant seizure type(s), and etiology [2]. Three broad classes of epilepsy include genetic generalized epilepsy (GGE; formerly idiopathic generalized epilepsy), focal epilepsy, and epileptic encephalopathy, though it should be noted that there are many specific epilepsy syndromes within each class (Table 1).

**Table 1:** Examples of epilepsy syndromes

Major class	Examples of specific syndromes
Genetic generalized epilepsy (GGE)	Juvenile myoclonic epilepsy (JME)
	Childhood absence epilepsy (CAE)
	Generalized epilepsy with febrile seizures plus (GEFS+)
Focal epilepsy	Temporal lobe epilepsy
	Autosomal dominant focal epilepsy with auditory features (ADPEAF)
Epileptic encephalopathy	Ohtahara syndrome
	Dravet syndrome
	West syndrome

The causes of epilepsy are diverse. Non-genetic or acquired etiologies account for 20–30 % of cases and include stroke, head injury, and tumor. In the remaining cases, genetics is thought to play a significant role. In fact, it has been recognized since the time of Hippocrates that epilepsy is, at least in part, genetic. Modern evidence for genetic factors comes from twin studies, family studies, and the identification of single-gene disorders resulting in epilepsy syndromes. Studies in twins show an excess of disease concordance in monozygotic twins compared to dizygotic twins for most types of epilepsy. In a large Australian cohort, generalized epilepsies showed the highest concordance (~80 %); focal epilepsies have a lower (36 %) but still significant concordance [3]. Family studies reveal that the overall recurrence risk for epilepsy in first-degree relatives of affected individuals is 2–5 % [4, 5], and at least one study has shown that there is similar increased recurrence for family members of probands with either generalized or focal epilepsy [6]. Finally, large multiplex families in which epilepsy segregates in an autosomal dominant manner have been used to identify linkage regions and causative genes in several different epilepsy syndromes [7].

Despite longstanding knowledge that epilepsy has a strong genetic component, it was not until 1995 that the first gene for a form of epilepsy was identified: mutations in the alpha 4 subunit of the nicotinic acetylcholine receptor, *CHRNA4*, were identified in a large family with autosomal dominant nocturnal frontal lobe epilepsy (ADNFLE) [8]. Since that time, multiple genes in which mutations cause epilepsy have been discovered [9, 10]. While many epilepsy genes encode ion channel subunits, several non-channel genes encoding proteins important for brain development have been recently discovered as well.

## Copy Number Variants and Human Disease

Copy number variants (CNVs) are large (>1 kb) deletions or duplications of DNA. CNVs can contain zero, one, or many genes and have been increasingly recognized as an important source of both normal genetic variation and pathogenic mutation. There are two major classes of CNVs: recurrent and non-recurrent. Recurrent CNVs are deletions and duplications that occur as a result of non-allelic homologous recombination (NAHR) at meiosis due to a predisposing sequence architecture: 50 kb to 10 Mb of unique DNA flanked by duplicated blocks of sequence that are >10 kb with >95 % sequence identity [11]. Examples of recurrent CNVs associated with neurological or neurodevelopmental disorders include duplications of 17p12 causing Charcot–Marie–Tooth type IA [12], deletions of 15q11-q13 in Prader–Willi and Angelman syndromes [13, 14], and deletions of 7q11 causing Williams–Beurens syndrome [15]. Because of the mechanism by which they are generated, recurrent CNVs in two unrelated individuals with the same disorder have nearly identical breakpoints.

Non-recurrent CNVs occur throughout the genome, but the breakpoints are not consistent. While recurrent CNVs are generated by aberrant recombination, non-recurrent CNVs are often due to errors at replication. There are several mechanisms for the generation of non-recurrent breakpoints that have been described, many of which involve microhomology—few to several identical base pairs at each breakpoint [16]. Non-recurrent CNVs can be simple, where a stretch of DNA is simply cut out of its original location and the ends rejoined, or complex, in which a deletion may be accompanied by insertion or duplication of DNA at the breakpoints for example. It is rare to find two or more patients with the same non-recurrent CNV. However, comparison of overlapping CNVs in similarly affected patients often reveals a “smallest region of overlap” that can highlight one or a few genes as primarily responsible for the phenotype. Examples include the discovery of *CHD7* as the gene for CHARGE syndrome [17], *EHMT1* as the critical gene in 9q34 deletions (Kleefstra syndrome) [18], and *MBD5* in 2q23.1 deletions [48].

Genome-wide identification of CNVs became efficient with the introduction of array comparative genomic hybridization (CGH) and single nucleotide polymorphism (SNP) microarrays. These technologies allow the detection of submicroscopic CNVs that are too small to be recognized by routine karyotype analysis. Since the introduction of these technologies, the rate of discovery of submicroscopic rearrangements in both affected and unaffected individuals has increased dramatically. Early application of CGH and SNP arrays in control cohorts produced a “CNV landscape” in unaffected individuals [19–27]. The first large disease cohorts to be systematically studied were patients with intellectual disability (ID), followed by autism and schizophrenia [28, 29]. Comparison of CNVs in patients to those in controls combined with the identification of similar CNVs in multiple affected individuals led to the discovery of novel, disease-associated CNVs. Systematic CNV discovery in patients with epilepsy followed, and there has been steady progress that provides new insight into the genetics of epilepsy and related disorders. The remainder of this review will focus on CNV discovery and characterization in patients with epilepsy and recommendations for clinical testing.

## Non-Recurrent CNVs in Epilepsy

The importance of non-recurrent CNVs in epilepsy has actually been known for some time. For example, techniques such as quantitative PCR and multiplex ligation-dependent probe amplification (MLPA) have been used to detect single- and multiple-exon deletions and duplications in known genes such as *SCN1A* [30, 31]. However, qPCR and MLPA are assays directed at specific locations in the genome, which means CNVs elsewhere will not be detected.

Real advances in CNV discovery came from the application of genome-wide investigations using array CGH or SNP microarrays. Heinzen and colleagues performed CNV discovery in a large cohort of 3812 patients with primarily focal epilepsy syndromes and identified an excess of large (>1 Mb) deletions in affected individuals, the majority of which were seen in one individual each [32••]. We performed array CGH studies in 517 patients with various types of epilepsy (primarily generalized); ~5 % of patients carried a non-recurrent CNV that affected at least one gene and was not seen in controls [33]. In a study of 102 patients with epilepsy with or without other neurodevelopmental abnormalities, 23/102 individuals had at least one non-polymorphic CNV [34]. Investigation of patients with epileptic encephalopathy syndromes also confirms the role of non-recurrent CNVs in severe epilepsies [35•]. Together, these studies confirm that CNVs are important contributors to the genetics of broad classes of epilepsy.

Because non-recurrent CNVs are rare and often unique, it can be difficult to interpret the clinical significance of any given CNV. Several criteria can be used, including size, gene content, presence or absence in control studies, and inheritance [36]. While a CNV in a single patient may be difficult to interpret, by collecting multiple patients with overlapping non-recurrent CNVs, it becomes possible to determine a critical region and, sometimes, a single critical gene for a given condition. This approach has been successful in highlighting several novel epilepsy genes. For example, Depienne and colleagues [37] performed array CGH on a series of patient with a Dravet-like syndrome and identified a single patient with a deletion involving *PCDH19* on chromosome X. Sequence analysis of the gene in additional patients revealed sequence mutations and established the gene as a cause of epilepsy restricted to females with ID. Similarly, rare reports of deletions involving the *GRIN2A* gene highlighted the gene as a potentially important gene [38]. Indeed, we and others have identified mutations in *GRIN2A* in 5–20 % of patients with epilepsy syndromes associated with language deficits (epilepsy aphasia syndromes) [39, 40, 41•]. We recently identified a patient with a large deletion of 15q26 (reported in [42•] ). Comparing the deletion in our patient to other 15q26 deletions in the literature highlighted a single gene in the smallest region of overlap: *CHD2*. Using high-throughput targeted sequencing of *CHD2* in 500 patients with epileptic encephalopathy, we identified de novo pathogenic mutations in 1.2 % of cases [43]. Additional studies of overlapping rearrangements published in large disease cohorts, as well as smaller case reports, are likely to yield important causative genes.

## Recurrent Deletions in Epilepsy

A major—and somewhat surprising—advance in the field of epilepsy genetics has been the discovery of recurrent CNVs in patients. The importance of recurrent CNVs as causes of ID syndromes, such as Prader–Willi, Angelman, Smith–Magenis, and velocardiofacial syndromes, has been known since the 1980s. More recently, genome-wide CGH in large cohorts of patients with ID led to the identification of several novel recurrent microdeletion syndromes [29, 44]. The first study that highlighted the importance of CNVs in the genetic etiology of epilepsy was the discovery of recurrent 15q13.3 deletions in patients with generalized epilepsy [45••]. The 15q13.3 microdeletion (chr15: 31,000,000–32,500,000, hg19) was first described in patients with ID, but it was noted that most patients also suffered from seizures [46]. This observation led to a collaborative effort to determine the frequency of the deletion in a cohort of 1,223 patients with generalized epilepsy, most of whom did not have ID or other neurodevelopmental abnormalities. Indeed, 12/1,223 (1 %) patients carried a 15q13.3 deletion compared to 0/3,699 control individuals [45••]. Several subsequent studies confirmed this finding [33, 47, 48], establishing the deletion as one of the most prevalent genetic risk factors for GGE with an estimated odds ratio of 68 (29–181) [47].

Two other recurrent deletions have been firmly associated with epilepsy. De Kovel and colleagues investigated a cohort of 1,234 individuals with GGE and 3,022 controls for recurrent CNVs [49]. In addition to 15q13.3 deletions, they found recurrent deletions at 16p13.11 (chr16: 15,500,000–16,300,000, hg19) and 15q11.2 (chr15: 22,800,000–23,100,000, hg19) in 0.5 and 1 % of patients, respectively, representing a significantly increased frequency compared to controls. Heinzen and colleagues performed CNV genotyping in 3,812 individuals with epilepsy, most of which presented with a focal epilepsy syndrome [32••]. Deletions of 16p13.11 were also enriched in patients compared to controls (23/3,812 vs 0/1299). While 15q11.2 deletions were identified in 24/3,812 patients, there was not a significant enrichment compared to the frequency in controls (3/1,299). In a study of 517 patients with various types of epilepsy, we identified five patients each with deletions at 15q11.2, 15q11.3, and 16p13.11, again emphasizing the importance of each of these as frequent genetic susceptibility factors in epilepsy [33]. Of note, in an investigation of 315 patients with epileptic encephalopathy, there were no occurrences of 15q13.3, 16p13.11, or 15q11.2 deletions [35•], suggesting a different genetic architecture for this class of disorders.

## Blurring the Lines: Shared Genetics of Neurodevelopmental Disorders

Each of the three epilepsy-associated recurrent deletions must be regarded as a risk factor for disease. In each case, the deletion may be de novo or inherited from an affected or unaffected parent. Importantly, all three deletions also confer risk for other neurodevelopmental disorders. As described above, deletions of 15q13.3 were first identified in patients from an ID cohort [46]. The deletion is also enriched in patients with schizophrenia [50, 51••] and is seen in patients with autism spectrum disorder and non-specific developmental delays [52, 53]. Similarly, deletions of 16p13.11 and 15q11.2 are also associated with a wide range of neurodevelopmental and neuropsychiatric conditions [32••, 54–57]. Interestingly, the 15q13.3 deletion appears to confer risk specifically for generalized forms of epilepsy—though present in 0.5–1 % of most GGE cohorts, it was not reported in >3,000 patients with focal epilepsy syndromes [32••].

Perhaps not surprisingly, patients with one of the epilepsy-associated recurrent deletions may present with a more severe phenotype than expected. This is especially true for GGE, which is not typically characterized by other neurocognitive deficits. Muhle and colleagues [58] identified 4/570 patients with various types of epilepsy who carried a 15q13.3 deletion. Detailed phenotype analysis revealed that all patients had absence epilepsy as well as some degree of ID. Similar features were described in two other families segregating the 15q13.3 deletion [59]. More recently, a systematic comparison of the frequency of recurrent CNVs in patients with GGE compared to patients with GGE and ID showed that patients with “dual disability” are more likely to carry one of the three epilepsy-associated CNVs than patients with GGE without other features [42•]: 10 % of the GGE + ID patients had one of the three recurrent CNVS compared to ~3 % of patients with GGE but no ID. Other recurrent CNVs that are associated with neurodevelopmental disorders are also found in patients with epilepsy, though not as frequently as the three deletions discussed above [28, 29, 60]. Examples include deletions and duplications of 1q21.1, 22q11.2, and 16p11.2.

## Who Should be Tested?

The role of CNVs in the genetic etiology of epilepsy has been clearly established, and diagnostic testing by chromosome microarray should be considered in this population. There is a clear consensus that chromosome microarray testing should be the first-line test in the diagnosis of patients with neurodevelopmental disorders or multiple congenital anomalies [36]. Given the overlapping genetic etiologies of a broad range of neurodevelopmental disorders, patients with epilepsy that is associated with other findings such as ID, autistic features, or developmental delays should be tested. Indeed, for GGE with ID, the diagnostic yield will be 10 % or greater. Similarly, patients with brain malformations or other congenital abnormalities and patients with epileptic encephalopathy without a clear diagnosis should undergo CNV testing. The yield of CNV testing in patients with epilepsy and no other features may be lower, but a CNV involving a known epilepsy gene would be an important diagnostic finding.

## Conclusions

The role of CNVs in the genetic etiology of epilepsy is now well established. Both recurrent and non-recurrent CNVs have been identified in most major classes of epilepsy. In some cases, CNVs are highlighting the shared genetic susceptibility for a range of neurodevelopmental and neuropsychiatric conditions. It is clear that the identification of disease-associated CNVs will lead to improved diagnosis and prognosis counseling. Recurrence risk counseling will remain complicated for CNVs with broad effects but is nevertheless an important consideration for families. Finally, as patients with shared genetic etiologies of epilepsy are identified, studies of genotype–phenotype correlation, natural history, and therapeutic response to specific anti-epileptic drugs can be performed, which will lead to improved long-term care and outcomes for patients.
